# Economic effects of the COVID-19 pandemic on entrepreneurship and small businesses

**DOI:** 10.1007/s11187-021-00544-y

**Published:** 2021-09-12

**Authors:** Maksim Belitski, Christina Guenther, Alexander S. Kritikos, Roy Thurik

**Affiliations:** 1 University of Reading, Henley Business School, Reading, UK; 2 ICD Business School, IGS-Groupe, Paris, France; 3 WHU, Otto Beisheim School of Management, Vallendar, Germany; 4 German Institute for Economic Research (DIW Berlin), Berlin, Germany; 5 University of Potsdam, Potsdam, Germany; 6 IZA, Bonn, Germany; 7 IAB, Nuremberg, Germany; 8 Montpellier Business School, Montpellier, France; 9 Erasmus School of Economics, Rotterdam, Netherlands

**Keywords:** Small businesses, Entrepreneurs, COVID-19 pandemic, Economic effects, C14, H43, L25, L26, J68

## Abstract

The existential threat to small businesses, based on their crucial role in the economy, is behind the plethora of scholarly studies in 2020, the first year of the COVID-19 pandemic. Examining the 15 contributions of the special issue on the “Economic effects of the COVID-19 pandemic on entrepreneurship and small businesses,” the paper comprises four parts: a systematic review of the literature on the effect on entrepreneurship and small businesses; a discussion of four literature strands based on this review; an overview of the contributions in this special issue; and some ideas for post-pandemic economic research.

## Abstract (plain language summary)

Responding to COVID-19 involves not just shielding small business jobs, supporting entrepreneurship, and raising government debt but also creating productive entrepreneurship and resilient location-specific entrepreneurial ecosystems. The COVID-19 pandemic is an unprecedented challenge for small businesses that also brings new market opportunities. The papers in this special issue of Small Business Economics Journal aim to shed light on the economic effects of the COVID-19 pandemic by looking at the macro- and microeconomic effects on entrepreneurship and small businesses as well as the role of financial support policies and well-being in both developed and developing countries. Future research should focus on the role of digitization and financial mechanisms supporting small businesses during crises.

## Introduction

Epidemics and pandemics do not just come and go, they impact the economy and society. For example, the epidemic in the early 1830s, when France (and other parts of central Europe) was hit hard by cholera with hospitals overwhelmed by patients whose ailments doctors could not explain (O'Sullivan, 2021). While the epidemic wiped out at least 3% of Parisians in the first month, it would contribute to an industrial revolution in France. It also increased political instability and social disparity, with the city’s poor being hit hardest by the pandemic, while the wealthier used their savings and resources to relocate from pandemic-impacted cities and reduce their interactions with the community (Economist, 2021).

The Spanish flu affected most of Europe and the USA in 1918. While it infected 500 million people—about a third of the world’s population at the time—it killed between 20 and 50 million people across four successive waves, including some 675,000 Americans (History.com, 2020). The enforcement of various restrictions varied across the cities and countries: the New York City Health Commissioner, for example, ordered businesses to open and close on staggered shifts to avoid overcrowding on the subway (History.com, 2020). In the USA and Europe, businesses were forced to shut down because so many employees were sick. Several authors demonstrate that the Spanish flu pandemic gave way to new businesses, with start-ups booming from 1919 in the middle of the pandemic onward (Beach et al., 2020; Karlsson et al., 2014).

The COVID-19 pandemic presents an unprecedented challenge in many ways. First, it threatens millions of people’s lives all over the world. It has already taken a death toll of almost four million people worldwide, as of the end of June 2021 (Worldometers, 2021). At the same time, the social distancing guidelines, taken to contain the virus, affected the service sector in particular, an area where physical proximity often matters and a sector that depends more on micro and small businesses than the manufacturing sector.

Therefore, COVID-19 directly affected self-employed individuals more than employed individuals (Kritikos et al., 2020) and small businesses more than large businesses, both in Europe and the USA (Digitally Driven, 2020, 2021).

A survey conducted by NBER of more than 5800 small businesses in the USA found that 43% of small firms were expected to be closed by December 2020 (Bartik et al., 2020). Small firms in hospitality, retail, personal services, entertainment, and the arts were most affected (Bartik et al., 2020). A survey conducted by the Connected Commerce Council of more than 5016 European small and medium-sized businesses carried out in November–December 2020 found that practically all SMEs were affected, with an average 20% decrease in sales and a 16% decrease in customer base (Digitally Driven, 2021).

Barrero et al., (2020: 17) demonstrate for the USA that, “temporary layoffs and furloughs account for 77% of gross staffing reductions in the first months of crises in the United States,” while the Financial Times (2020) reports that, “more than 3 m Americans filed for first-time unemployment benefits during a first week of May 2020, taking the number of applications for the first three months of the lockdown to 33.5 million. The number of working business owners in the United States plummeted from 15.0 million in February 2020 to 11.7 million two months later in April” (Fairlie, 2020). In the UK, the unemployment rate surged to its highest level since 2017 as the pandemic continued to affect jobs (Thomas, 2020). In the long term, the COVID-19 pandemic is expected to become a cleansing process and a large reallocation shock (Caballero and Hammour, 1991) for firms of different sizes and industries.

Governments throughout the world responded with support initiatives. In the USA, the largest program providing funds to small businesses is the Paycheck Protection Program (PPP) with a volume of $650 billion during the early stages of the pandemic (Bhutta et al., 2020). The Small Business Administration (SBA)–administered program provided loans to small businesses through banks, credit unions, and other financial institutions with the goal of keeping small businesses open and retaining employees on the payroll (Fairlie & Fossen, 2021). In the UK, the government implemented the Coronavirus Job Retention Scheme (CJRS) (popularly known as “the Furlough” scheme) for waged workers. The CJRS covers 80% of employee salaries up to a maximum of £2500 per month. More than 8.7 million jobs were furloughed at an estimated total cost of around £60 billion (Yue & Cowling, 2021). After initially ignoring the 4.6 million self-employed, the UK government announced the Self-Employment Income Support Scheme, which awarded grants of 70% of average monthly trading profits calculated from tax returns for 2018 and 2019. This scheme only applied to those self-employed who earned less than £50,000 in profit for the relevant period (Yue & Cowling, 2021). The measures supported by the German government intended to protect businesses and start-ups affected by the COVID-19 crisis include taxation support, state-supported short-time work compensation schemes, improved measures at guarantee banks, loans and special programs provided by KfW (Kreditanstalt für Wiederaufbau) (PWC, 2020), and an emergency aid that offers one-off lump sum payments to self-employed facing substantial revenue declines (Block et al., 2020).

In China, measures started in February 2020 when Chinese central bank unblocked extensions or renewals of loans to companies and announced a reduction in the banks’ mandatory reserve ratio. The government presented a package to support the digitalization of SMEs in the context of the crisis. A wide range of policy measures was announced for SMEs at the regional level in China, including deferred tax payments for SMEs, reducing rent costs, waiving administrative fees, subsidizing R&D costs for SMEs, social insurance subsidies, subsidies for training and purchasing teleworking services, and additional funding to spur SME loans (KPMG, 2020). The 2020 GEM report mentions that 54 national governments made emergency policy decisions and actions in response to the COVID-19 pandemic (GEM 2020). Unprecedented amounts of state aid were channeled into propping up economies around the globe.

Despite the deployment of administrative, fiscal, and monetary tools to counter the fall in employment and demand, it seemed unlikely that these measures will be enough to attain a full offset. The response to COVID-19 requires both top-down and bottom-up approaches, e.g., government and private initiatives to support productive entrepreneurs, instead of dying industries and failing firms.

The shock of the pandemic may further increase inequality in at least two ways: First, female owners of small businesses faced a 35% higher probability of experiencing income losses than their male counterparts with the gender gap among the self-employed being largely explained by the fact that women disproportionately work in industries that are more severely affected by the COVID-19 pandemic (Graeber et al., 2021). Second, the consequences of the COVID-19 pandemic may be more pronounced for minorities in developed (Fairlie and Fossen, 2021) and developing countries (Maliszewska et al., 2020; Pereira & Patel, 2021).

More efficient and productive incumbents are likely to grow, with new businesses and industries emerging. The new “Never-Meet-in-Person Era” will change industries, impacting large and small firms in certain industries, such as transport, hospitality, arts and entertainment, and personal services. The weight of hybrid firms, platform-based firms, and platform-matchmakers in the global economy will grow rapidly (Kenney & Zysman, 2020).

The emergence of digital technologies has significantly reduced the economic costs of data—search, storage, computation, transmission—and enabled new economic activities during the COVID-19 pandemic and a change in lifestyle. Since the start of the pandemic, small and large firms, able to create a platform-based ecosystem, have become a force of “creative destruction,” value creation, and value appropriation (Acs et al., 2021).

The big issue is how the shock and the resulting recession will affect firms, large and small, young and mature, family and non-family firms, community-embedded small firms, and platform-based blitz-scalers not only in the short term but also the mid- and long terms. Will this be different than for any other exogeneous shock?

The potential consequences for businesses may include but are not limited to closed premises, reduced operating hours, job cuts, supply chain disruptions, jeopardizing the R&D processes, cessation of operations, business model changes, loss of key customers, and restrictions on products/services.

News stories highlight the millions of layoffs triggered by the pandemic and lockdown (Barrero et al., 2020), while they also relate to examples of large-scale hiring. For example, on April 18, 2020, Walmart reported that it had hired 150,000 new employees, with plans to hire 50,000 more (Nassauer, 2020). Fidelity Investments and Fifth Third Bancorp have also been on “hiring sprees,” and hires through Zoom eliminated the worry to be spotted during a job interview lunch by current employers. Will this be the beginning of a new revolution toward large multinational corporate structure, away from micro and small businesses? Businesses may have had different experiences from responding to the previous recessions and other pandemics but can these lessons be useful for small and large firms to respond to COVID-19?

Therefore, the objective of this special issue is to examine the economic effects of the COVID-19 pandemic on entrepreneurship and small businesses as well as help to promote research and economic implications relevant to understanding the nature of the pandemic shock, consequences, and opportunities for SMEs and large firms in the short- and long-term perspectives more broadly.

The present introduction to the special issue is organized as follows. It consists of four parts: a systematic review of the literature on the effect of COVID-19 on entrepreneurship and small businesses; a discussion of four literature strands based on this overview; an overview of the contributions in this special issue; and some ideas about the post-pandemic economic research, organized according to four avenues.

## Systematic literature review

We start our analysis by performing a “systematic literature review” (Tranfield et al., 2003). It is a reliable and efficient method of identifying and evaluating a sizeable literature volume and is widely used in business research (Verma & Gustafsson, 2020). The advantage of this method is that it allows for capturing all existing studies on the topic, to incorporate quantitative, qualitative, and mixed-method studies, as well as to identify the state of knowledge regarding theories, special entities, and fields of study.

**Fig. 1:**
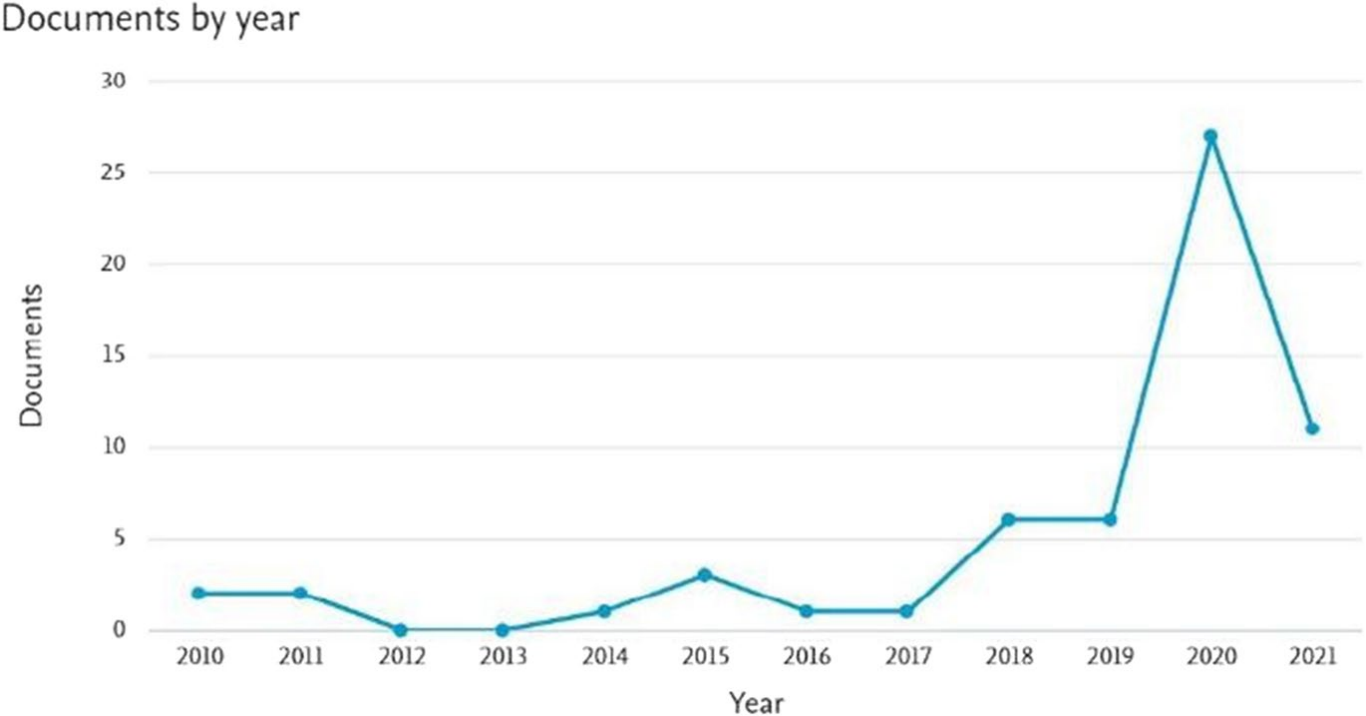
Timeline of publications on small business and the Spanish flu. Note: 2021 is an incomplete year since the research was done in May of that year

Based on this systematic and comprehensive literature review, we investigate research gaps and identify areas that require further research using the Scopus and Web of Science database, taking our lead from prior systematic literature reviews of Rousseau et al. (2008) and Verma and Gustafsson (2020). Before moving to the systematic literature review on the effect of COVID-19 pandemic on small business and entrepreneurship, we wanted to find out whether there is prior research on the economic effects of historic pandemics, such as the Spanish flu. Therefore, we use the period of 50 years, which resulted in only 60 publications, related to the effect of Spanish flu on small businesses. Interestingly, most papers on the effect of the Spanish flu on small business were published during the COVID-19 pandemic (see Fig. 1). Researchers from the USA, UK, and Canada have led this field of research.

Our next, and main, step was to review the literature on the economic effects of COVID-19 on small businesses and entrepreneurship. We used the period from December 2019 to June 2021 because it corresponds to the pandemic period. We included all articles, data sets, early-access publications, and data studies in English, yielding 3607 published pieces. Once we applied the selection criteria, including only articles published in international peer-reviewed journals, in English and the area of study, the number of publications dropped to 1789. The distribution of articles by field of science is as follows: social sciences (29.3%), business management (22.6%), economics (12.9%), environmental sciences (10.9%), energy (8.8%), organizational studies (2.3%), arts and humanities (2.0%), psychology (1.9%), and other (9.30%).

**Fig. 2:**
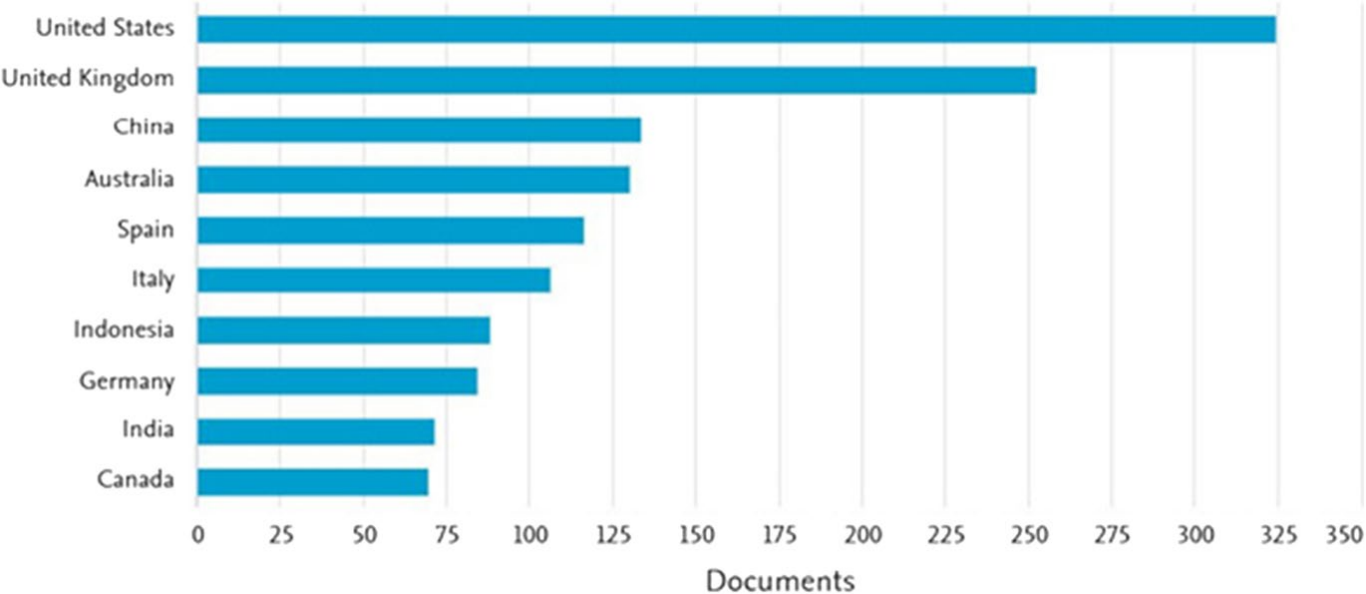
The region of the publications on small business and COVID-19

In the third stage, we used the field of research exclusion criteria with the aim of retaining publications from relevant fields such as business economics, management, social sciences, and economics. Most of the publications come from the USA, China, and the UK (see Fig. 2).

We excluded the BIOSIS Citation Index, BIOSIS Previews, Medline, Zoological Record, and FSTA. This means that we just kept the Web of Science and Scopus databases, yielding to 285 papers. Based on the keywords, text, and abstracts from these 285 papers, we created the visualization network to identify the themes related to the impact of COVID-19 on small businesses using VOSviewer. Co-word analysis applies text-mining techniques to the papers’ titles, abstracts, keywords, and text. Co-word connections allow for identifying and combining multiple co-occurrences and keywords in the same paper, as well as determining the relationship between different keywords (Verma & Gustafsson, 2020).

**Fig. 3:**
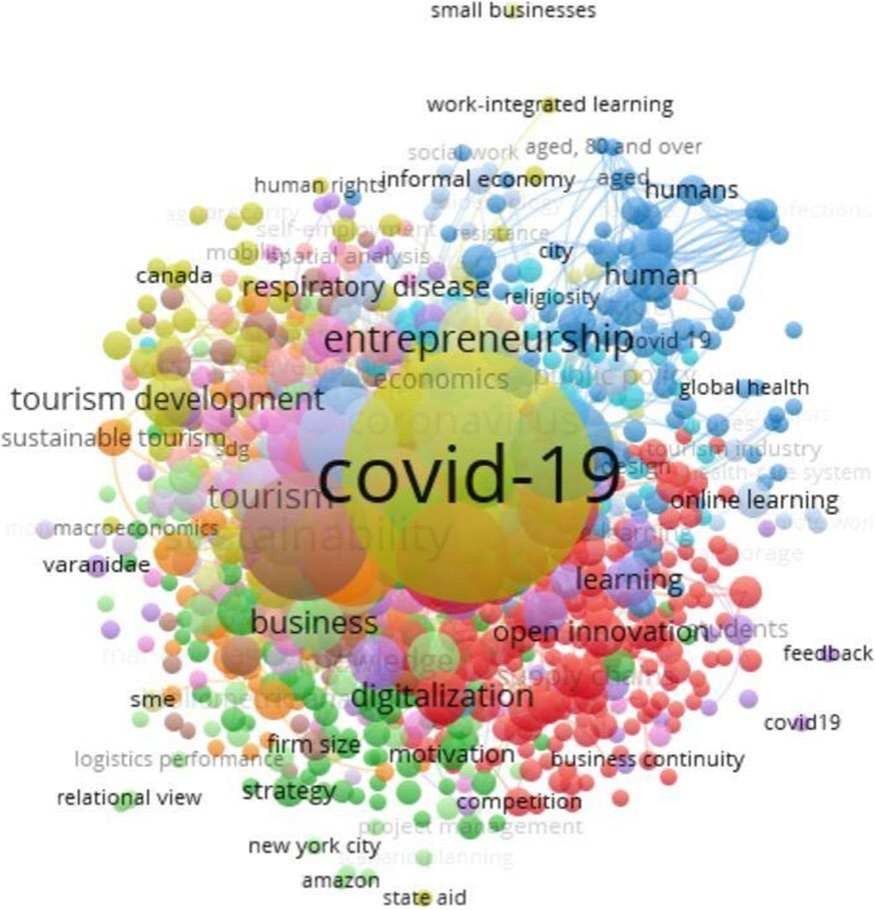
The keywords network visualization

The outcome of the systemic literature review resulted in a keywords network visualization that required (i) selecting the patterns of topics and (ii) clustering topics theories: digitization and open innovation, resilience and disaster, knowledge creation and learning (dynamic capabilities), including industry effects (e.g., healthcare, information technology, tourisms) (Fig. 3). The theories were identified by reading all the abstracts and keywords of the 285 papers. These four theories are further explained in the next section and will be matched to the papers that comprise this special issue. We note that a clear discrimination between these literatures is not always possible.

## Theories and contributions

Based on the systematic literature review, this section describes how four literatures can be used by scholars to better understand and explain the economic effects of the COVID-19 pandemic on small business across different countries, firm sizes, and the severity of the crisis. First, there is disaster theory literature, which focuses on the financial and physical resources enabling small firms to be more resilient during crises. A body of literature stresses the importance of community-based networks and the role of social capital in helping small businesses to respond to disasters (Bin & Edwards, 2009; Torres et al., 2019).

Torres et al. (2019) investigate small business owners’ response to natural disasters and catastrophes through the lens of resources and social capital, drawing a line between resilient small businesses that not only remain operating but also thrive after a disaster and those exiting. Evidence focusing on small businesses shows that they widely engage in disaster relief for their community (Bin & Edwards, 2009), clarifying that in addition to governments, entrepreneurs and small businesses also become active (Markman et al., 2019). Post-disaster business resilience is the product of many complex decisions resulting from the interaction of individuals, families, businesses, and communities (Marshall & Schrank, 2014).

Second, responses to crises and exogeneous shocks is at the heart of resilience theory. The origins of the resilience concept in the business literature go back to Staw et al. (1981) and Meyer (1982). Both authors draw upon variation–selection–retention mechanisms posited by evolutionary theory (Campbell 1965) and develop very different propositions regarding how organizations respond to external shocks. Staw et al. (1981) introduce a theory on how negatively framed situations lead to risk avoidance in the form of “threat-rigidity effects.” Meyer (1982) extends the resilience framework by studying hospital responses to an unexpected doctors’ strike or “environmental jolt,” contradicting the proposition by Staw et al. (1981) that an external threat automatically places an organization at risk.

Resilience takes place over time and is related to the recovery of individuals, businesses, communities, and institutions. Most studies consider post-disaster business resilience as a binary stage of open or closed businesses (Marshall & Schrank, 2014). By capturing measures and processes that contribute to small business resilience as a disaster response, Tugade and Fredrickson (2004) provide real world examples, while Torres et al. (2019) emphasize the role of community and support to entrepreneurs in a post-shock period.

Research on resilience and post-disaster management literature began to comment that there are few avenues to detect whether or not an entrepreneur had “resilience potential,” prior to demonstrating a resilient or non-resilient response (Linnenluecke et al., 2012). Furthermore, researchers argue that more attention should be devoted to the period of detecting a threat and activating firm’s response. Conceptualization of organizational resilience broadly fall in three categories: (1) resilience as an outcome, (2) resilience as a process, and (3) resilience capabilities (Bullough et al., 2014; Duchek, 2020).

In the post-COVID world, agile and resilient new businesses will be able to take advantage of their entrepreneurial orientation and find opportunities in the upheaval that the pandemic has caused globally (Zahra, 2020). In an environment characterized by high volatility and uncertainty, the importance of the firms’ dynamic capabilities (DC) to integrate resources in recognizing new opportunities is also further heightened (Battisti & Deakins, 2017). The role of DCs and the role of resilience (Bergami et. al, 2021; Bullough & Renko, 2013; Bullough et al., 2014) are differentiators between not just the survival and failure of small businesses and entrepreneurs and also the speed with which new ventures are able to learn, both determining their growth and survival in the long term (Zahra, 2020).

Third, there is a literature on the role of knowledge creation and absorptive capacity in addressing the negative effects of disasters and crises. Dynamic capabilities (DC) are the key concept underlying absorptive capacity as the antecedent organizational and strategic routines by which managers alter their resource base—acquire and shed resources, integrate them together, and recombine them—to generate new value-creating strategies (Eisenhardt & Martin, 2000; Grant, 1996). Teece et al., (1997: 516) defines DCs as “the firm’s ability to integrate, build, and reconfigure internal and external competences to address rapidly changing environments. Dynamic capabilities thus reflect an organization’s ability to achieve new and innovative forms of competitive advantage given path dependencies and market positions.”

Managing uncertainty tends to be the new normal for many companies around the world (i.e., climate change, COVID-19), thus stressing the importance of creating competitive advantage and improving dynamic capabilities that are so important for small business (Arend, 2013) and that seem to be the only antidotes to uncertainty during the COVID-19 pandemic (Flammer & Ioannou, 2020).

The role of dynamic capabilities was brought forward by Priyono et al. (2020) in their analysis of how small businesses cope with environmental changes due to the COVID-19 pandemic by pursuing the business model transformation with the change in dynamic capabilities related to adaptation of digital technologies and digital skills.

Dynamic capabilities, which became even more relevant in the digital era (Li et al., 2016), enable small businesses to adopt digital tools more quickly and efficiently. This enables stronger response to the COVID-19 pandemic. For example, Audretsch and Belitski (2021) demonstrate how European small businesses adopt digital technologies and develop strategic, managerial, and digital skills to increase their efficiency.

The DC theory could be relevant in the response to the volatility, velocity, and criticality of COVID-19 effects (Obal & Gao, 2020), for instance, by redeploying salespeople to virtual rather than physical sales calls. The literature on dynamic capabilities could draw on prior research in times of high turbulence but is sparse and focuses mainly on financial crises. For example, Fainshmidt and Frazier (2017) and Makkonen et al. (2014) find a disconnect between pre-crisis settings and the types of DCs most useful during crisis.

Bartik et al. (2020) and Kuckertz et al. (2020) suggest how government initiatives help businesses cope with the COVID-19 pandemic. A further cluster of papers use information gathering surveys (e.g., Bartik et al., 2020; Fairlie, 2020; Kritikos, et al., 2020) and case studies (Kuckertz et al., 2020; Robinson & Kengatharan, 2020). There is a lack of research on the intersection of the pandemic and DCs.

It is important to understand the boundary conditions explaining whether DCs can benefit small businesses compared to larger firms. Prior research suggests the existence of a positive feedback loop that results in firms with the largest initial capability endowments generating more new capabilities. Taken together, despite prior research on DC in small businesses (Arend, 2013; Fainshmidt & Frazier, 2017), only a few studies deal with the role of firm size in determining DCs and in response to the COVID-19 shock.

The fourth strand of literature is related to digitization and the role of digital capabilities in adopting new business models, responding to uncertainty, and developing resilience. Behavior of rapidly growing small businesses depends on their business models (Hennart, 2014; Kuratko et al., 2020), and the role of digitally enabled firms and business models is important in times of volatility (Li et al., 2016; Vadana et al., 2019). The role of digital capabilities is expected to grow in importance for entrepreneurship and small business research and practice during and after the COVID-19 crisis. Digital capabilities will be able to change business models and introduce business model innovation (Clauss et al., 2019). In the entrepreneurship literature, entrepreneurial growth remains an oft-neglected topic of research, as only a few studies (Asemokha et al., 2019; Child et al., 2017) shed light on the dynamics of business models and growth or performance in entrepreneurship.

There is still a gap with respect to understanding which DCs need to be developed for firms to respond to opportunities of COVID-19, such as digitalization and business model change (Seetharaman, 2020).

Works on digitization in small businesses analyze the implementation of business intelligence as part of their efforts to increase competitiveness in a highly dynamic business environment. A better understanding of the adoption levels of innovation by small businesses is relevant due to the important contribution of small businesses to both employment generation and economic growth (Audretsch et al. 2021b).

Studies commissioned by Google in the USA in 2020 and in Europe in 2021 demonstrate that the so-called Digital Safety Net has empowered millions of small businesses to shift resources, modify business plans, and continually evolve throughout the pandemic (Digitally Driven, 2021). The COVID-19 pandemic threatened small businesses globally, but their use of digital tools has acted as a “Digital Safety Net” and saved many of them (Digitally Driven, 2020, 2021).

## Papers in the present special issue

The papers in this special issue can be divided into four strands by the unit of analysis, policy implications, and the literature used. These strands can be connected to the four literatures distinguished in the previous section. The first strand reveals the macro-economic effects of Covid-19 on entrepreneurship, small businesses, and the role of digital technologies in changing work routines of entrepreneurs, which relates to the literature on disaster management and the role of digital tools and capabilities. The second strand touches upon the economic and socio-psychological impact of the COVID-19 pandemic on entrepreneurship building on resilience literature and literature on the role of dynamic capabilities, in addition raising the issues of inequality and the effects of COVID-19 in developing and developed economies. The third strand deals with financial support to small businesses and entrepreneurship, building on the literature that addresses the negative effects of disasters and crises as well as macroeconomic responses to shocks. Finally, the fourth strand discusses the effect of various policy and well-being issues for small businesses during COVID-19 drawing on resilience and disaster theory literature.

The first strand contains three papers. Addressing the macroeconomic effects of COVID-19 on the way of living and working, a study of Zhang et al. (2021) “Working from Home: Small Business Performance and the COVID-19 Pandemic” focuses on working from home as an opportunity rather than an activity that leads to frustration, loneliness, and worries about the future (Banerjee & Rai, 2020). In this paper, working from home appears to be an opportunity to improve small businesses’ performance in the COVID-19 crisis. The authors built a theoretical framework based on firm profit maximization using daily and weekly data to demonstrate that working from home impacts the industrial structure and peoples’ work behavior.

A study by Meurer et al. (2021) demonstrates how entrepreneurs can use alternative support sources of communication and business, such as online communities, raising the question of how support is created in such spaces. Drawing on an affordances perspective, the authors investigate how entrepreneurs interact with online communities and base their qualitative analysis on conversation data (76,365 posts) from an online community of entrepreneurs on Reddit during the COVID-19 pandemic. The findings draw out four affordances that online communities offer to entrepreneurs (resolving problems, reframing problems, reflecting on situations, refocusing thinking and efforts), resulting in a framework of entrepreneurial support creation in online communities.

Altogether these two papers demonstrate how small businesses and individual entrepreneurs can adjust to new business conditions by working from home, developing new business models, and seeking social support to leverage the negative impact of the COVID-19 pandemic.

The study of Pedauga et al. (2021), “Macroeconomic Lockdown and SMEs: The Impact of the COVID-19 Pandemic in Spain,” takes a macroeconomic perspective to empirically test the role of small business in the economy. The authors use a financial social accounting matrix to distinguish between the direct and indirect effects that are transferred from micro, small, medium, and large firms to the rest of the economy during the COVID-19 pandemic. The authors explore the sequence of reactions associated with shocks that arise from the COVID-19 lockdown to small businesses using a structural model for the Spanish economy and identifying the role of businesses of different sizes for the gross domestic product (GDP). Interestingly, small businesses “explain” 43% of the gross domestic product and two-thirds of the unemployment decline caused by the COVID-19 pandemic.

The second strand of studies in this special issue examines the economic and non-economic impact on small business performance of the COVID-19 pandemic. The study of Grözinger et al. (2021) on “The Power of Positivity: Organizational Psychological Capital and Firm Performance During Exogenous Crisis” investigates how psychological capital in businesses impacts performance and creative innovation through organizational citizenship behavior, solidarity, and cooperation. The authors use structural equation modelling and regression analysis on 379 small businesses to demonstrate that psychological capital positively influences creative innovation and thus performance during crises. This research contributes to the organizational behavior approach of the small business literature by showing that psychological resources of small businesses can strengthen performance in times of crisis and help to prepare for future shocks.

The study by Torrès et al. (2021a), “Risk of Burnout in French Entrepreneurs During the COVID-19 Crisis,” discriminates between three sources of burnout: the threat of becoming ill, having to stay at home due to the lockdown, and having to file for bankruptcy due to the economic downturn. They use seven data sets of French entrepreneurs with a temporal comparison of averages and two data sets of French entrepreneurs with a cross-sectional analysis of individuals. They show that the risk of burnout increased during the pandemic, that all three factors play important roles, and that the financial threat is the dominant one. These findings call for the extension of entrepreneurial support systems beyond the financial by also involving an “entrepreneurship care” aspect, which includes telephone support, webinars, mental help facilities, and other support measures.

The study by Kalenkoski and Wulff Pabilonia (2021), called “Impacts of COVID-19 on the Self-employed,” uses monthly panel data from the Current Population Survey in the USA and examines the initial impacts of COVID-19 on the employment and hours of unincorporated self-employed workers. The authors find that effects become visible in March 2020 as voluntary social distancing started, peaked in April during the complete shutdown, and were slightly smaller in May. They conclude that self-employed married mothers were hit hardest and were even forced out of the labor force to care for children. Moreover, remote work and working in an essential industry mitigate some of the negative effects on employment and hours worked.

Pereira and Patel (2021) in their study, “Is the Impact of COVID-19 More Severe on Self-employed of Colour? Large Scale Evidence from Brazil,” complement prior research on self-employed from racial minority groups and use resilience theory to explain how minority self-employed in Brazil responded to the COVID-19 pandemic with lessons for other developing countries (e.g., Sri Lanka) (Robinson & Kengatharan, 2020). The paper extends the argument that minorities may face greater adversity from the COVID-19 pandemic in the USA and other developed countries (Buheji et al., 2020), while there is little evidence that minority self-employed in a developing country are also significantly affected in the context of the COVID-19 pandemic.

The third strand of studies brings together the role of financing for entrepreneurship and small businesses in crises and a variety of support tools. Studies in this part discuss the role of financial support and other government programs to respond to economic disruption. Various support policies were developed and provided by governments all over the world in response to address their small businesses’ financing needs. In a paper by Liu et al. (2021), “SMEs’ Line of Credit under the COVID-19: Evidence from China,” the Chinese SMEs’ financing responses to the outbreak of COVID-19 are examined. The study shows the supportive role of Chinese state-owned banks on small businesses’ lines of credit. These policy instruments can be broadly categorized into loan guarantees, direct lending to small businesses, grants and subsidies, and equity instruments. Interestingly, there are considerable differences in supporting small businesses’ financing policies between countries. For example, in the USA, European Union, the UK, and China and Russia, policies to support small businesses during the pandemic were a commonplace. Brazilian and Indian government provided little support to small business.

The study of Fairlie and Fossen (2021), “Did the Paycheck Protection Program and Economic Injury Disaster Loan Program Get Disbursed to Minority Communities in the Early Stages of COVID-19?,” examines the effect of the US federal government response to help small businesses—the Paycheck Protection Program (PPP) and the related Economic Injury Disaster Loans (EIDL). The program’s stated goal is helping disadvantaged groups. The authors provide the first detailed analysis of how the 2020 PPP and EIDL funds were disbursed across minority communities in the country. The authors find a positive relationship between PPP loan receipt per business and the minority share of the population or businesses, although funds flowed to minority communities later than to communities with lower minority shares. This study acknowledges the importance of financial support through PPP loans of minority communities as a share of the population. The important evidence is that the EIDL program, both in numbers per business and amounts per employee, was positively distributed to minority communities. This is the first study about how loans and advances from these programs were distributed between minority and non-minority communities.

Another study by Atkins et al. (2021), “Discrimination in Lending? Evidence from the Paycheck Protection Program,” adds to our understanding of the role of race in loans made through the Paycheck Protection Program (PPP). Expanding the paper of Fairlie and Fossen (2021), the authors argue that the historical record and PPP program design choices made it likely that many Black-owned businesses received smaller PPP loans than White-owned businesses: Black-owned businesses received loans that were approximately 50% smaller than observationally similar White-owned businesses. Interestingly, the effect is marginally smaller in areas with more bank competition and disappeared over time as changes to the PPP program were implemented allowing for entry by fintechs and other non-traditional lenders.

The study by Block et al (2021), “The Determinants of Bootstrap Financing in Crises: Evidence from Entrepreneurial Ventures in the COVID-19 Pandemic,” investigates the measures that entrepreneurial ventures undertake to preserve liquidity. The authors build on prior research on bootstrap financing as an important enabler for the growth of resource-constrained early-stage ventures. Their work fills the gap about the use of bootstrap financing during COVID-19, during which the preservation of liquidity is particularly salient. The determinants of bootstrap financing are embedded into a “necessity” human capital perspective and an “opportunity” cost perspective. The analyses are based on data of 17,046 German entrepreneurial ventures.

The study of Dörr et al. (2021), “Small Firms and the COVID-19 Insolvency Gap,” focuses on fiscal policy in rescuing companies short of liquidity from insolvency. The authors show that, in the first months of the crisis, the small businesses that are the backbone of Germany’s economy benefited from large and mainly indiscriminate aid measures. The authors estimate the extent to which the policy response induced an insolvency gap and analyze whether the gap is characterized by firms that were already struggling before the pandemic. They also examined whether this insolvency gap differs with respect to firm size and find that the gap was larger for smaller firms. The theoretical contribution of the paper is in translating Schumpeter’s theory of the cleansing effect in economic crises into an empirical assessment by estimating the size of a policy-induced insolvency gap using firm-specific credit rating data combined with information on insolvency filings.

The fourth strand of studies represents a variety of micro and macro public support and well-being programs aiming to mitigate the negative effects of the COVID-19 crises.

The Lastauskas (2021) study, called “Lockdown, Employment Adjustment, and Financial Frictions,” examines businesses’ employment adjustments after the imposition of stringent lockdown in March 2020. It uses monthly administrative data and takes value-added tax payment changes as a proxy for the demand shock. The main finding is that all businesses in the manufacturing sector reduced employment more if they had uncovered tax liabilities before the lockdown. Among small businesses, those in the real estate and the service sectors downsized more rapidly. While employment changes are rather modest, this early evidence points to the importance of addressing liquidity needs and specific pre-conditions among capital-intensive and services businesses to avoid employment losses.

The Belghitar et al. (2021) study, “When the rainy day is the worst hurricane ever: the effects of governmental policies on SMEs during COVID-19,” examines the impact of COVID-19 on 42,401 UK small businesses and how government intervention affected their capability to survive the pandemic. The results show that, without governmental mitigation schemes, 59% of UK small businesses report negative earnings and that their residual life is reduced from 164 to 139 days. This analysis demonstrates that government financial support may reduce the number of small businesses with negative earnings and allows extending the residual life for small businesses with negative earnings up to 194 days. Block et al. (2020), who analyze the first emergency aid program in Germany, find similar effects among German businesses hit by the crisis. Interestingly, the study of Belghitar et al. (2021) highlights that—in contrast to Block et al (2020)—those industries that were worst hit by COVID-19 are not those that benefited the most from the government support scheme. The possible reason is that the government scheme does not differentiate between firms that do or do not deserve support.

Finally, the study of Braunerhjelm (2021) deals with macro-economic stabilization policies and discusses that targeting aggregate demand may not suffice to mitigate the comprehensive effects of the COVID-19 crisis. Entitled “Rethinking Stabilization Policies: Including Supply-side Effects and Entrepreneurial Processes,” it suggests that a more active role for fiscal policies is needed and presents a modified framework for stabilization policies, giving an extended role to supply-side measures and emphasizing policies that can promote entrepreneurial processes and knowledge upgrading efforts. Aligning policies at the micro- and macro-levels can be expected to counteract economic downturns more efficiently as the potential for long-term growth is enhanced. Such a redirection of stabilization policies is argued to strengthen the competitive standing of both firms and individuals.

## Future research

There are many discussions and arguments proclaiming that nothing in business will be left unchanged: in the post-COVID world, there will be opportunities for entrepreneurs to embark on creating new products and services, with novel business models and business routines arising that are different from traditional ones (Janssen et al., 2021). Changes in (the perception of) well-being, the way of consuming, in the way of filtering out the resilient and the agile, the adoption of new digital technologies and learning skills, and much more will all contribute to something that some call the “new normal.” Below, we contribute to this discussion with respect to four dimensions of future research, all connected to the contents of this special issue, initially sparked by our discussions with authors and referees during the online paper-development-workshop organized by the University of Reading on November 20, 2020: caution is warranted as all suffer from a certain degree of speculation.

### Long- and short-term economic effect of COVID-19

The results of several papers in this special issue demonstrate that investigating the long-term effects induced by the policy responses to COVID-19 on turnover, productivity, innovation, and entrepreneurship in developed countries is needed. However, future research may also want to demonstrate a wider economic, political, and societal challenge, including inequality and poverty, unemployment within poor countries, and the gap between rich and poor countries (Bartik et al., 2020; Robinson & Kengatharan, 2020).

Real wages in certain sectors may rise, such as tourism, hospitality, and restaurants, as the disease reduces the supply of workers, leaving survivors in a stronger bargaining position.

The macro- and microeconomic effects of the COVID-19 shock are different between small and large firms as well as between the self-employed and incorporated business. Smaller businesses are typically disadvantaged in their ability to capture the opportunities that crises have created. It is important to research further the role of local and national governments, public organizations, civil society, and other stakeholders in mitigating the effect of crises.

Forming partnerships between small and large firms, the role of open innovation and knowledge spillovers may emerge as an important conduit for entrepreneurship and for mitigating the effects of COVID-19. Particularly interesting is the dynamics of so-called science, technology, engineering, and mathematics (STEM)–related jobs in the long term.

Further insights are needed to understand economic and psychological drivers of innovation during crises. While previous research demonstrates that context matters (Audretsch et al., 2021a; Welter, 2011; Welter et al., 2019), the context of a crisis is a compelling, yet understudied, one. Welter et al. (2019) outlines three recent and overlapping waves of contextualization in the entrepreneurship field and shows that the discussion has moved from challenging the Silicon Valley model by considering the why, what, and how of entrepreneurship (first wave) to considering more subjective elements in enactment of contexts (second wave), through broadening the domain of entrepreneurship research (third wave).

To quantify the effect of the COVID-19 lockdown on economic activity, it may be possible to consider the links between all three waves (Welter et al., 2019) at the idiosyncratic level and their aggregate impact. It is probably not just sectoral issues and those issues related to the labor market or economic growth that play a role, but also deeper mental issues (Torrès et al., 2021a).

### The use of digital technology, competencies, and robots

Digital skills trends seem to be interacting with the pandemic and its social, political, economic, environmental, and demographic tensions, combining to accelerate the reconfiguration of production and service systems. This reconfiguration of existing skills and adoption of digital skills not only affects employment trends, but also the way we work and experience our mental and physical health, perhaps even long after the crisis is over.

The role of digital technology has significantly increased under COVID-19. For instance, digital technologies affected the way firms do head-hunting during COVID-19 as well as how products and services are manufactured and delivered. During disease outbreaks—Ebola in 2014–2016 and COVID-19 in 2019, among others—the adoption of robot and digital tools accelerates, especially when the health impact is severe and associated with potential economic losses or economic crises.

Entrepreneurship in the post-pandemic world will further fuse with the digital economy. This will take the form of entrepreneurs increasingly selling products on digital platforms, using digital tools like TikTok for marketing and relying on platforms such as Kickstarter for funding. Moreover, we believe that entrepreneurs will further seek to use peers in online communities to develop opportunities, get assistance with problems, and find collaborators. The key implication is that, while entrepreneurs in the past have often physically worked side by side to develop their business locally, in the future such bounds will play a diminishing role. One can start a business in Ghana, work with a programmer in Indonesia, find a marketing specialist in Paris, secure funding over Kickstarter, and sell the product through a digital platform. In other words, COVID-19 fosters the transition of the entrepreneurial economy into a digital, disembodied economy. The next big technology to be adopted at large scale is likely to be 5G. The large-scale use of artificial intelligence is being pushed but may not be relevant until 2025 at the earliest. Quantum computing is also being pushed, but not likely to affect small businesses before 2030.

All small businesses must be prepared for the “new normal” of a digitally driven economy (Meurer et al., 2021). Many are well positioned, but others feel uncertain due to challenges accessing capital, tools, and training, as well as with measuring success. During the pandemic, so-called advanced small businesses invested more than twice as much money in digital tools than the so-called uncertain small businesses (Digitally Driven, 2021). The working environment changed fundamentally with the digitalization and flexibilization of work receiving a considerable boost. These changes probably make companies more resilient to future shocks.

Even though the self-employed initially were hit harder by the COVID-19 pandemic than larger firms in the USA and Europe (Digitally Driven, 2020, 2021), there is reason to be optimistic because, for the millions of SMEs that still lack skills, technology, and resources, adopting digital tools is within reach with the right mindset, strategy, access to world-class digital technologies, and training. As the working world has become more flexible, it is likely that mixed forms of remote and physical working (especially in teams) will become accepted in the future. However, we also learned that remote work cannot sufficiently replace personal encounters in all cases. Therefore, we believe that society and the working world will learn to appreciate such personal encounters again and that these will be valued differently in the future. Future research may need to better understand the role personal encounters and skills, which, along with new technology, will be valued more in the future.

### Financing for entrepreneurship

As witnessed by several contributions in the present special issue, there are many promising avenues for research regarding what drives the financing of entrepreneurial activity during and after the COVID-19 crisis. For example, we would expect that entrepreneurial motivation may play an important role, along with networks of venture capital and angel investors. A significant share of solo self-employed individuals start their businesses out of necessity (Block et al., 2015; Caliendo & Kritikos, 2019; De Vries et al., 2020; Zwan et al., 2016). As policymakers want more high-growth ventures to recover from the crisis, their interest in opportunity-driven entrepreneurs may grow. Human and social capital including networks for entrepreneurship may be important for sourcing entrepreneurial financing. Finally, research should also analyze performance effects and investigate whether and how various sources of finance, beyond bootstrapping during the COVID-19 crisis, may impact long-term entrepreneurial performance, survival, and high growth (Audretsch et al., 2021b).

Financial support policies are important for supporting small businesses and individual entrepreneurs with the mechanisms and the extent of such support being substantially different between OECD and non-OECD countries. Thus, understanding the causes and consequences of SME financing policies in the COVID-19 era would be intriguing and pivotal for both academic researchers and policymakers. Future research could also examine whether and how the institutional and development stage heterogeneities shape the policy differences related to stakeholders, unit of financing, and form of financing (e.g., grants, loans, equity). In that sense, the pandemic is a natural experiment.

A criticism of the financial support programs is that often there was no data collected on applications for loans that were denied (Fairlie & Fossen, 2021). This is an important piece of information that should be collected for future research on public support to small businesses and entrepreneurs to gauge demand and unmet need for these loans, in particular by minority businesses in developed and developing countries. As in the case of the USA, PPP and EIDL funds were allocated to support businesses, and it is crucial to track who receives funding and how it helps small businesses to become more resilient and grow during the crisis.

During the first phase of the pandemic, massive government support slowed firm exits. However, it may be argued that the resources were not spent efficiently and that public support mechanisms slowed down industrial dynamics. Hence, an important challenge for the post-pandemic world is to revitalize entry rates and stimulate technology adaption while also encouraging the adoption of new business models that restore productivity and growth beyond pre-crisis levels. In this context, research in industrial dynamics may help to contribute to the existing long-run challenges faced by modern societies such as digitization, decarbonization, and sustained prosperity.

Looking ahead, government and policymakers may want to design financial policy interventions that dampen the impacts of the pandemic on small businesses. Future research should focus on direct policies, like zero-interest loans, subsidies, and grants. According to Liu et al. (2021), in this special issue, the measures should target subgroups, firms that heavily rely on supply chains, and small businesses without stable bank relationships.

Understanding the effects of the interplay between liquidity support, on the one hand, and temporary adjustments to insolvency regimes, on the other, will provide an important lesson from the COVID-19 crisis. Further research may focus on the interplay of these two instruments as it is assumed that they may discourage struggling firms from exiting the market.

### Non-economic effects of the COVID-19

An increasing number of studies in the entrepreneurship literature analyzes to what degree entrepreneurs’ mental health influences their activities. Further studies about the perception of burnout or general mental health issues, with a focus on experiences during the COVID-19 pandemic across more countries, industries, and fields, could expand what we know about the response of entrepreneurs during crises and how negative effects (e.g., burnout) could be leveraged.

COVID-19 put a large strain on entrepreneurs, who experienced an unprecedent shock to their businesses (Torrès et al., 2021b) Without being able to meet physically with investors and clients, some entrepreneurs had to scale down their businesses; others closed their business, and solo entrepreneurs were left more isolated than before. The COVID-19 pandemic has likely been detrimental to the mental health of entrepreneurs. The pandemic forced entrepreneurs to reflect on the importance of their mental health and to actively seek and establish coping techniques. Some entrepreneurs experiencing failure may decide that entrepreneurship is not for them, but we expect that those who continue their entrepreneurial career found ways to cope with high stress levels. For instance, such entrepreneurs will use “time boxing” to become more productive, meditate regularly, or use digital tools to connect with peers. These entrepreneurs will likely also focus more on balancing their working and private lives by creating a working situation that suits their social needs. In that sense, some of the entrepreneurs who suffered during the pandemic may come back mentally stronger and more resilient.

The lockdown likely led to frustration, loneliness, and worries about the future (Kritikos et al., 2020), which are also risk factors for mental illnesses (Banerjee & Rai, 2020). Future research can focus on the impact of lockdowns and quarantine on small businesses as well as on the link between lockdowns, psychological effects (Brooks et al., 2020), and entrepreneurship (Shepherd, 2020). Results of future investigations could inspire entrepreneurs to search for novel, more sustainable, and more social forms of entrepreneurship, better understanding failures and successes of small businesses. This knowledge, which is often informal and tacit, represents a source of wealth for dealing with new forms of crisis (both health related and economic).

Protecting and supporting the health of small businesses and entrepreneurs during and after the COVID-19 pandemic is essential because they have a special role in the aftermath of crisis and in the anticipated post-pandemic boom. This aftermath may be predominantly dematerialized with a virtual mode of working and new norms of working from home. The climate and the green agenda would be a priority. A large part of business services would be contactless. Entrepreneurs’ health—both physical and mental—would be acknowledged and recognized as vital, both by the entrepreneurs themselves and by the policy makers.

However, given the length of school closures and the considerable reduction in the availability of childcare centers, the gender gap in entrepreneurship, which was identified at the beginning of this crisis, may widen in the post-pandemic period (Seebauer et al., 2021).

In general, economic inequality between and within nations is likely to also increase the likelihood of contracting the coronavirus and dying from it. Developing nations with weak healthcare systems and an inability to practice social distancing also account for the unequal impact. For people of low socio-economic status and economically disadvantaged people in developed countries, COVID-19 also poses higher risks of living in overcrowded accommodations increasing risk of illness (Patel et al., 2020). Racial and ethnic minorities experience higher death rates from COVID-19, which has also unequally affected urban residents and foreign migrants around the world. With the closure of schools, nurseries, and other childcare facilities for all but children of essential workers (Blundell et al., 2020), parents were typically left with the sole responsibility for caring for their children, including education, which particularly affected the survival of the self-employed. How these growing inequalities affect business dynamics will become an entire field of scholarly research and, hopefully, of compensating policy interventions.
